# Prevalence and association of *pks*^+^ *Escherichia coli* with colorectal cancer in patients at the University Malaya Medical Centre, Malaysia

**DOI:** 10.1371/journal.pone.0228217

**Published:** 2020-01-28

**Authors:** Thevambiga Iyadorai, Vanitha Mariappan, Kumutha Malar Vellasamy, Jane Wangui Wanyiri, April Camilla Roslani, Goh Khean Lee, Cynthia Sears, Jamuna Vadivelu

**Affiliations:** 1 Department of Medical Microbiology, Faculty of Medicine, University of Malaya, Kuala Lumpur, Malaysia; 2 University of Malaya Centre of Proteomics Research (UMCPR), University of Malaya, Kuala Lumpur, Malaysia; 3 Department of Medicine, Johns Hopkins University School of Medicine, Baltimore, Maryland, United States of America; 4 Department of Surgery, University of Malaya, Kuala Lumpur, Malaysia; 5 University of Malaya Cancer Research Institute, Kuala Lumpur, Malaysia; 6 Department of Medicine, Faculty of Medicine, University of Malaya, Kuala Lumpur, Malaysia; 7 Department of Oncology, Sidney Kimmel Comprehensive Cancer Center, Johns Hopkins University School of Medicine, Baltimore, Maryland, United States of America; Kurume University School of Medicine, JAPAN

## Abstract

***Escherichia coli*** (*E*. *coli*) from the B2 phylogenetic group is implicated in **colorectal cancer** (CRC) as it possesses a genomic island, termed **polyketide synthetase** (*pks*), which codes for the synthesis of **colibactin**, a genotoxin that induces DNA damage, cell cycle arrest, mutations and chromosomal instability in eukaryotic cells. The aim of this study was to detect and compare the prevalence of *E*. *coli* expressing *pks* (*pks*^+^ *E*. *coli*) in CRC patients and healthy controls followed by investigating the virulence triggered by *pks*^+^ *E*. *coli* using an *in-vitro* model. Mucosal colon tissues were collected and processed to determine the presence of *pks*^+^ *E*. *coli*. Thereafter, primary colon epithelial (PCE) and colorectal carcinoma (HCT116) cell lines were used to detect cytopathic response to the isolated *pks*^*+*^ *E*. *coli* strains. Our results showed 16.7% and 4.3% of CRC and healthy controls, respectively were *pks*^+^ *E*. *coli*. Further, PCE displayed syncytia and cell swelling and HCT116 cells, megalocytosis, in response to treatment with the isolated *pks*^*+*^ *E*. *coli* strains. In conclusion, *pks*^+^ *E*. *coli* was more often isolated from tissue of CRC patients compared to healthy individuals, and our *in-vitro* assays suggest these isolated strains may be involved in the initiation and development of CRC.

## 3 Introduction

Globally, colorectal cancer (CRC) is the second most common type of cancer in females and third most common type of cancer in males with 694,000 fatal cases recorded in 2012. Similarly in Malaysia, the National Cancer Patient Registry reported CRC as the second most common cancer in both males and females with a total of 4,501 cases diagnosed and reported from 2008–2013 [1]. In general, CRC incidence is higher in developed countries as compared to underdeveloped countries but the burden of disease globally is rising including in middle income nations [2, 3]. Although genetic predisposition carries a significant risk for CRC, various studies have implicated environmental factors such as smoking, alcohol consumption, sunlight and ionizing radiation, organic and inorganic chemicals, viruses and bacteria, diet and obesity, hormone therapy and air and water pollution to play major roles in CRC incidence [4, 5]. Currently, there are ample of research being conducted in order to find the definite cause of CRC and based on the findings, researchers theorized that the bacteria present in the human colon might be linked to the development of carcinogenesis [6].

Typically there are trillions of commensal bacteria that co-exist in a human colon, including *Bacteroides*, *Fusobacterium*, *Escherichia*, *Clostridium*, *Lactobacillus*, *Bifidobacterium*, *Eubacterium*, *Peptococcus*, and *Veillonella* among others [7]. In general, anaerobes outnumber aerobes in the gut bacterial community and any form of disruption to the composition of this microbiota causes a condition known as dysbiosis [8]. Among the gut microbes, *Escherichia coli* (*E*. *coli*) is commonly isolated from both CRC patients and healthy controls; however more pathogenic strains are isolated from CRC patients as compared to healthy individuals [6], [9, 10]. These pathogenic *E*. *coli* strains are able to induce a specific cytopathic effect (CPE) known as megalocytosis when in contact with mammalian cells and this effect is dissimilar to other known toxins produced by *E*. *coli* such as cytolethal distending toxins (CDT) and cytotoxic necrotizing factors (CNF). Previous studies have demonstrated that the genes involved in CPE were found in a specific genomic island that is widely found only in the *E*. *coli* B2 phylogenetic group [11]. This genomic island, termed the polyketide synthetase (*pks*) island, is responsible for the expression of peptide-polyketide hybrid genotoxic-cyclomodulin (a non-ribosomal peptide synthetase and *pks*) referred to as colibactin (*clb*) [11], [12], [13]. Recent studies have shown that CRC patients more frequently harbor *pks*^*+*^ *E*. *coli* strains in their colonic mucosa as compared to non-cancerous patients [6], [12], [13]. The presence of *pks*^*+*^ *E*. *coli* induces double strand DNA breaks in mammalian cell lines, which consequently lead to cell cycle arrest followed by cell death [14]. All these outcomes indicate that the specific genotoxin from *E*. *coli* B2 phylogenetic group may play a significant role in colon carcinogenesis.

However, to date, there are no available data on the carriage of these *pks*^*+*^ *E*. *coli* amongst Malaysians. The aim of this study was to evaluate the presence, role and impact of *pks*^*+*^ *E*. *coli* in Malaysian CRC patients. Therefore, we report on the detection and comparison of colonic colonization of *pks*^*+*^ *E*. *coli* in Malaysian CRC patients and healthy controls from the University Malaya Medical Centre (UMMC), a tertiary teaching hospital of the University of Malaya, Kuala Lumpur. In addition, we also investigated the phenotypic effect of *pks*^*+*^ *E*. *coli* on human colonic epithelial cells through an *in-vitro* assay.

## 4 Materials and methods

### 4.1 Ethics statements

This study was part of collaborative research study between Johns Hopkins University School of Medicine, Baltimore, USA and University of Malaya, Kuala Lumpur, Malaysia. This study was approved and carried out in accordance with the recommendations by the Johns Hopkins Institutional Review Board and Medical Institutional Review Board and the University of Malaya Medical Center (UMMC) Medical Ethics Committee (Ethics reference number: 943.18) with written informed consent from all subjects. All subjects gave written informed consent in accordance with the Declaration of Helsinki.

### 4.2 Recruitment of colorectal cancer patients and healthy controls

A total of 48 adult (≥18 years old) CRC patients who underwent colon or rectal cancer resection between April 2014 and July 2015 at UMMC were prospectively recruited in this study. These CRC patients were subjects that had no history of inflammatory bowel disease (IBD) or CRC. Surgery was performed by either laparotomy or laparoscopy. Following the resection, colon tumor tissue and their matching non-malignant (non-tumor) tissues at the far edge of the resection were collected. In addition, 23 adult (≥18 years old) healthy controls who underwent colonoscopy at the UMMC endoscopy unit between March 2014 and August 2015 were also recruited in this study. The healthy controls were subjects with no previous history of IBD or CRC or any form of cancer. During the colonoscopy procedure, biopsies from normal-appearing colon were collected from both the right (proximal) and left (distal) colon. Samples obtained from the cecum and ascending colon up to the hepatic flexure were defined as proximal while those obtained from the transverse colon, splenic flexure, descending colon, sigmoid and rectum were defined as distal.

Both CRC patients and healthy controls consisted of male and female subjects from Malay, Chinese, Indian and other races recruited in this study as accordance to the inclusion and exclusion criteria as listed above, Prior to the procedures (both surgical and colonoscopy), informed consent was obtained from all patients (both CRC and healthy controls) and they were also requested to complete a questionnaire that consisted information regarding their socio-demographic data such as their gender, race, height and weight as well as their medical history which included list of daily medicine intake (if applicable), personal and family history of any form or cancer and/or CRC, alcohol consumption and smoking cessation (S1 Appendix). All the obtained samples were processed immediately upon collection.

### 4.3 Microbiological analysis

The fresh tissue samples from 48 CRC patients (48 tumor and 48 matching non-malignant tissue) and 23 normal colon biopsies from healthy controls (23 proximal and 23 distal biopsies) were cut into equal sizes of 4 mm diameter using a disposable biopsy punch and homogenized in Luria Bertani (LB) broth (Friendemann Schmidt, 28320) using a polypropylene tissue grinder pestle. The tissue homogenate was then incubated for 18 hours at 37°C (shaking at 180 rpm). Following incubation, the tissue homogenate was spread on MacConkey agar (selective agar) (Friendemann Schmidt, HCM017) and further incubated for 18 hours at 37°C. Single colonies (pinkish) were isolated from the agar plate for genomic DNA extraction.

### 4.4 Molecular analysis

DNA from single colonies isolated was extracted using QIAamp DNA Mini Kit (Qiagen, 51306) according to the manufacturer’s instructions. Concentration and purity of the DNA was measured using Nano Photometer (IMPLEN, Munich, Germany). The extracted DNA was used immediately or stored at -20°C until further used. Primers for the *E*. *coli* small subunit (*16S* rRNA) gene (reference) and *E*. *coli* colibactin (*clbB*) gene (target) were synthesized by Intergrated DNA Technologies (IDT) (Singapore). The *16S* rRNA gene was amplified by PCR using a primer set universal for *E*. *coli*: forward primer 5’-CATGCCGCGTGTATGAAGAA-3’ and the reverse primer 5’–CGGGTAACGTCAATGAGCAAA-3’, while the *clbB* gene was amplified by PCR using the following primer set: forward primer 5’-GCGCATCCTCAAGAGTAAATA- 3’ and the reverse primer 5’ -GCGCTCTATGCTCATCAACC-3’ [15, 16]. GoTaq® Green Master Mix (Promega Corporation, M8295) was used for the PCR reactions. DNA from *pks*^*+*^ *E*. *coli* (ATCC^®^ 25922^TM^) was used as a positive control and a negative control was prepared by substituting the DNA with nuclease free water. The GoTaq® Green Master Mix consisted of GoTaq® Flexi Buffer, MgCl_2_ solution, PCR Nucleotide Mix and GoTaq® DNA Polymerase. The final volume of the PCR reactions was 25 μl, comprised of the reagents from GoTaq® Green Master Mix, the primers (forward and reverse) and nuclease-free water. Upon amplification using a thermal cycler (Bio-Rad, CFX96 Touch^TM^ Real-Time PCR Detection System) (94°C ^5:00^ [94°C^0:30^; 60°C^0:45^; 72°C^0:30^]_35_ 72°C^2:00^), the PCR products were then evaluated and visualized on a 1% agarose gel (GeneMark, GA0001) containing SYBR® Safe DNA gel stain (Invitrogen, S33102). The expected product size of the 16S rRNA and *clbB* genes were 100 bp and 280 bp, respectively. Those samples positive for both 16S rRNA and *clbB* genes were acknowledged as *E*. *coli pks*^*+*^.

### 4.5 Cytopathic assays

The primary colon epithelial (PCE) cell line (T4056, ABM,) and colorectal carcinoma (HCT116) cell line (ATCC® CCL-247^TM^) were purchased and obtained directly from its respective company (ABM for PCE cell line while ATCC for HCT116 cell line) to be used in this study. PCE cell was isolated from a normal human colon section while HCT116 cell was obtained from CRC patient. Roswell Park Memorial Institute (RPMI) 1640 (Gibco, 31800–022) working media (10% fetal bovine serum (FBS), 1% L-glutamine, and 1% penicillin-streptomycin) was used to grow both the cell lines at 37 ºC with 5% CO_2_. The cells were seeded in 6 well tissue culture plates (Corning, 3596) at 2x10^5^ cells/well and incubated at 37 ºC with 5% CO_2_ for 24 hours prior to infecting them with the *E*. *coli* strains isolated from the CRC patients or healthy controls. During this step, antibiotics were removed from the RPMI media to prevent killing of the infecting bacteria.

To create the *E*. *coli* inoculum, a single colony of each *E*. *coli* strain (both *pks*^+^ and *pks*^-^) was inoculated into 5 ml of LB and incubated at 37°C for 24 hours with continuous shaking (180 rpm). The turbidity of the bacterial culture was then measured and adjusted to OD_600nm_ 0.1 and further incubated at 37°C with continuous shaking (180 rpm) until the culture reached mid-logarithmic phase of the growth. The bacterial cultures were then centrifuged at 7,500 rpm for 10 minutes and the recovered pellet re-suspended with RPMI media (without antibiotics). In preliminary experiments, the PCE and HCT116 cells were infected with *E*. *coli* (*pks*^+^ and *pks*^-^; MOI 1:100) strains and incubated at 37 ºC with 5% CO_2_ for a period of 24 to 72 hours to determine the optimal incubation period to detect cytopathic effect (CPE).

Microscopic analysis showed that the optimal incubation period for the detection of CPE was 48 hours for HCT116 and 72 hours for PCE (S1 Table). Subsequent assays were evaluated at this time points. After the respective incubation periods, the supernatant was discarded without disturbing the attached cells and left to air dry for about 30 minutes before fixing the cells with 99% methanol for 5 minutes followed by staining with diluted Giemsa stain (10%) for 10 minutes. After air drying the cells for about 30 minutes, the cells were viewed microscopically (Leica, Germany) for the presence or absence of CPE.

## 5 Results

### 5.1 Study subjects

**Fig 1:**
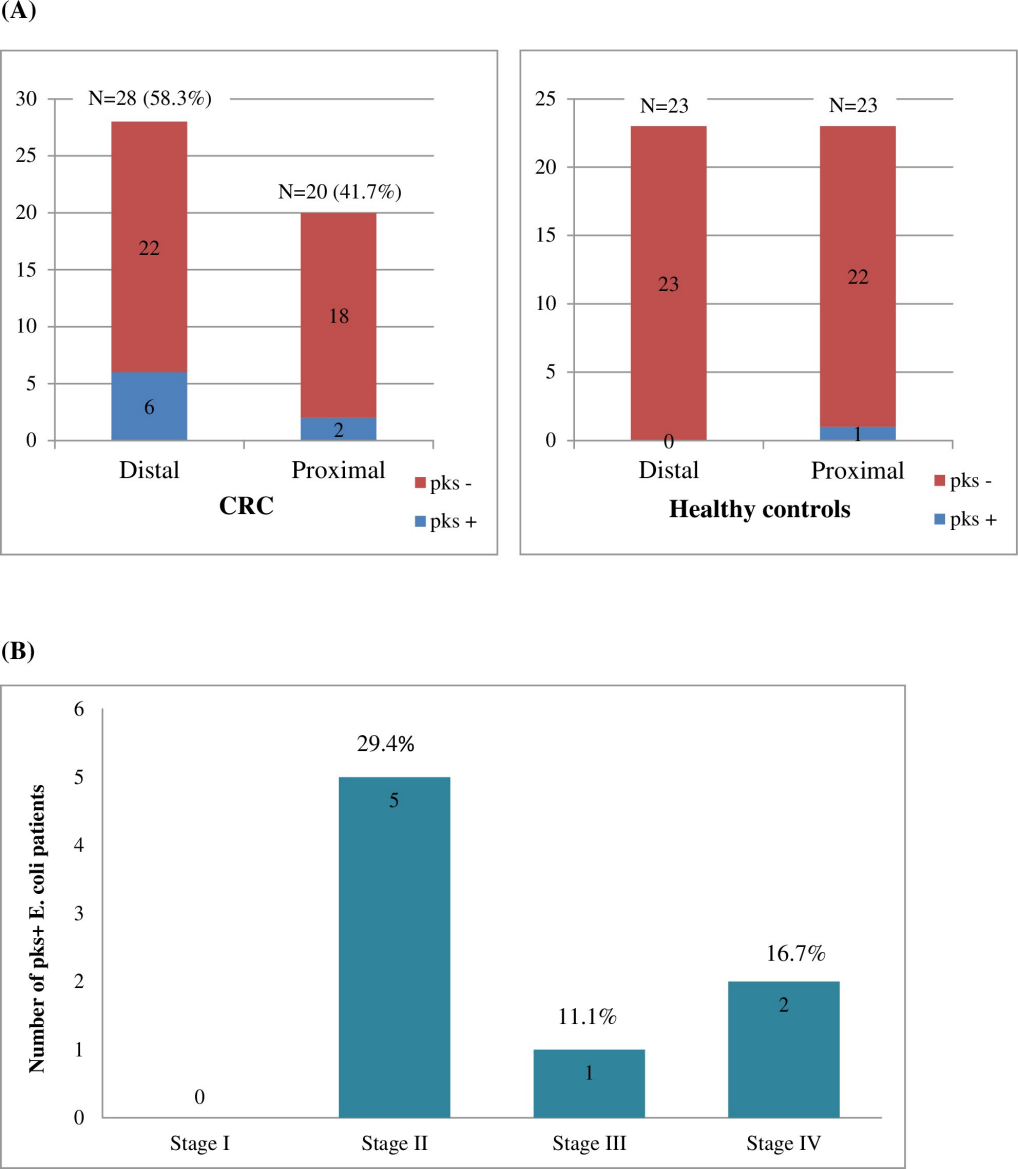
CRC patients and healthy controls’ *pks*^*+*^ *E*. *coli* status. (A) More *pks* positive cases in CRC from the distal side of the colon compared to the proximal side (B) The number of *pks* positive cases in CRC with different tumor stages.

In total, 71 human subjects (48 CRC patients and 23 healthy controls) were recruited in this study (Table 1). Among the 48 CRC patients and 23 healthy controls recruited, 52% and 47.8% were males, respectively. Additionally, the majority of both the CRC and healthy controls were of Chinese origin (66.7% and 69.6%, respectively). Of the 48 CRC patients, 28 (58.3%) had a distal colon tumor while the remaining 20 patients had a proximal colon tumor (Fig 1A). Ten (20.8%) CRC patients were diagnosed with Stage I cancer, 17 (35.4%) patients with Stage II cancer, 9 (18.8%) with Stage III cancer and 12 (25%) patients with Stage IV cancer. Tumors were staged according to the American Joint Committee on Cancer (AJCC - 6^th^ edition) classification for Tumor, Nodes and Metastases (TNM) staging [17].

**Table 1:** Characteristics of the study subjects.

Study Subjects	Healthy controls (n = 23) (%)	Colorectal cancer patients (n = 48) (%)
**Age, y, median (IQR)**	62 (51–72)	67.5 (60–74)
**Gender**		
** Male**	11 (47.8)	25 (52)
** Female**	12 (52.2)	23 (48)
**Race**		
** Malay**	3 (13)	10 (20.8)
** Chinese**	16 (69.6)	32 (66.7)
** Indian**	3 (13)	5 (10.4)
** Others**	1 (4.4)	1 (2.1)
**Histologic diagnosis**		
** Adenocarcinoma**	NA	-
** Stage I**	-	10 (20.8)
** Stage II**	-	17 (35.4)
** Stage III**	-	9 (18.8)
** Stage IV**	-	12 (25)

Data are presented as No. (%) unless otherwise specified

Abbreviation: NA, not applicable

All the data are not significantly associated (Correlation is considered significant at the 0.05 level– 2 tailed)

### 5.2 Presence of *pks*^*+*^ *E*. *coli* in CRC cases

**Fig 2:**
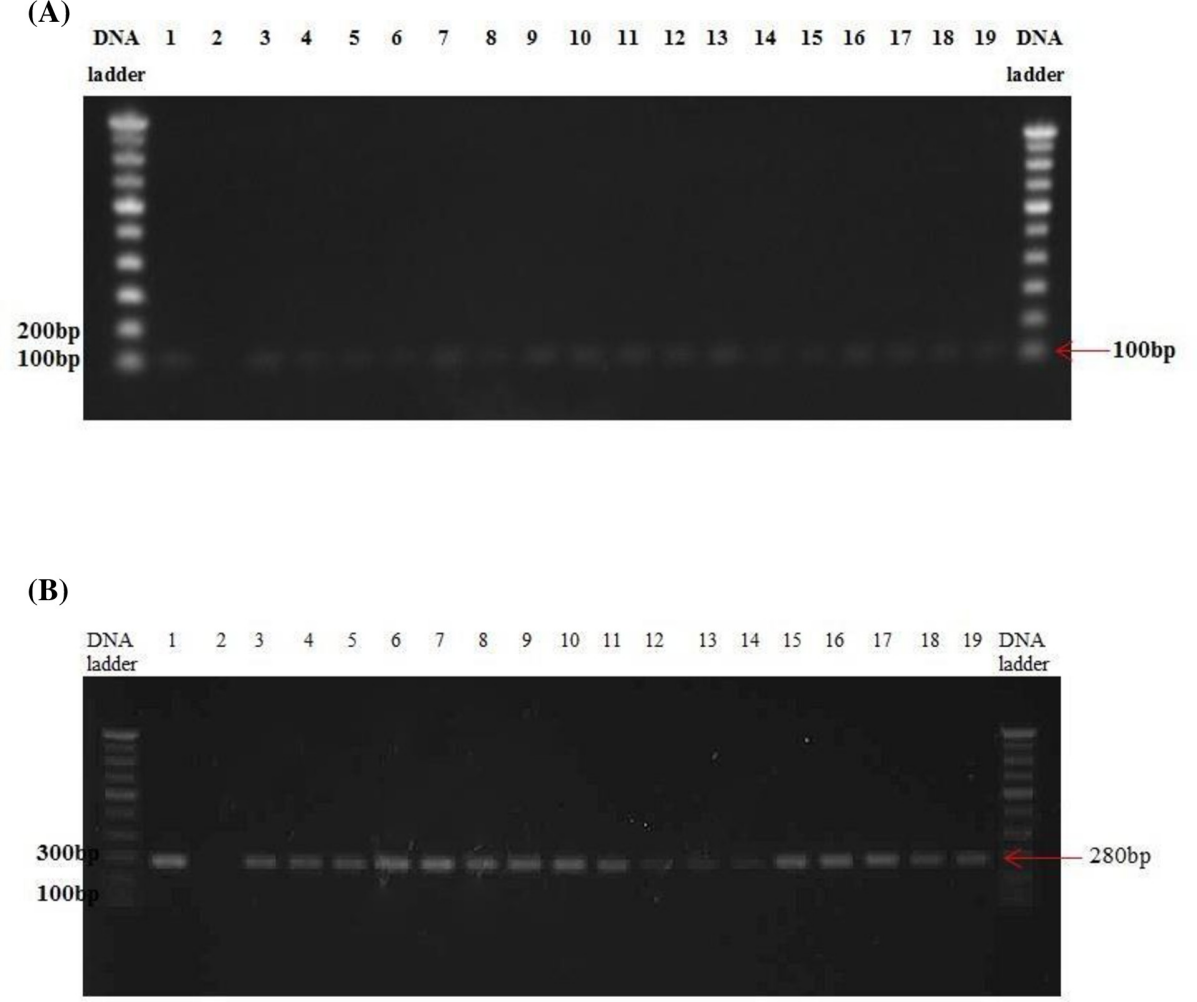
**PCR amplification;** (A) 16*s rRNA* (100 bp) *and (B) colibactin (clbB)* genes (280 bp) of CRC patients and healthy controls was visualized on 1% agarose gel. Lane 1 shows positive control (+) while lane 2 shows negative control (-), followed by lanes 3–18 showing positive bands from CRC patients and lane 19 shows positive band for healthy control.

Eight out of 48 (16.7%) CRC patients were found to be positive for *pks*^*+*^ *E*. *coli* compared to only one out of 23 healthy controls (4.35%) (χ^2^ = 2.1317, P = 0.144; Fig 2; S2 Table). When analyzed by the numbers of tissues examined, *pks*^*+*^ *E*. *coli* was detected on 1/26 colonoscopy biopsies from healthy controls and 16/96 tissue samples from CRC patients (χ^2^ = 6.1981, P = 0.01). All tumor:normal pairs were concordant for *pks*^*+*^ *E*. *coli* status; namely, if *pks*^*+*^ *E*. *coli* was isolated from the tumor, the organism was also isolated from the matching normal colon sample. Of the eight CRC patients colonized with *pks*^*+*^ *E*. *coli*, six presented with distal tumors while two had proximal tumors (Fig 1A). However, in the one healthy control, the *pks*^*+*^ *E*. *coli* was present only on the proximal colon biopsy, but not its respective distal colon biopsy.

### 5.3 Possible risk factors for patients with *pks*^+^ *E*. *coli*

**Fig 3:**
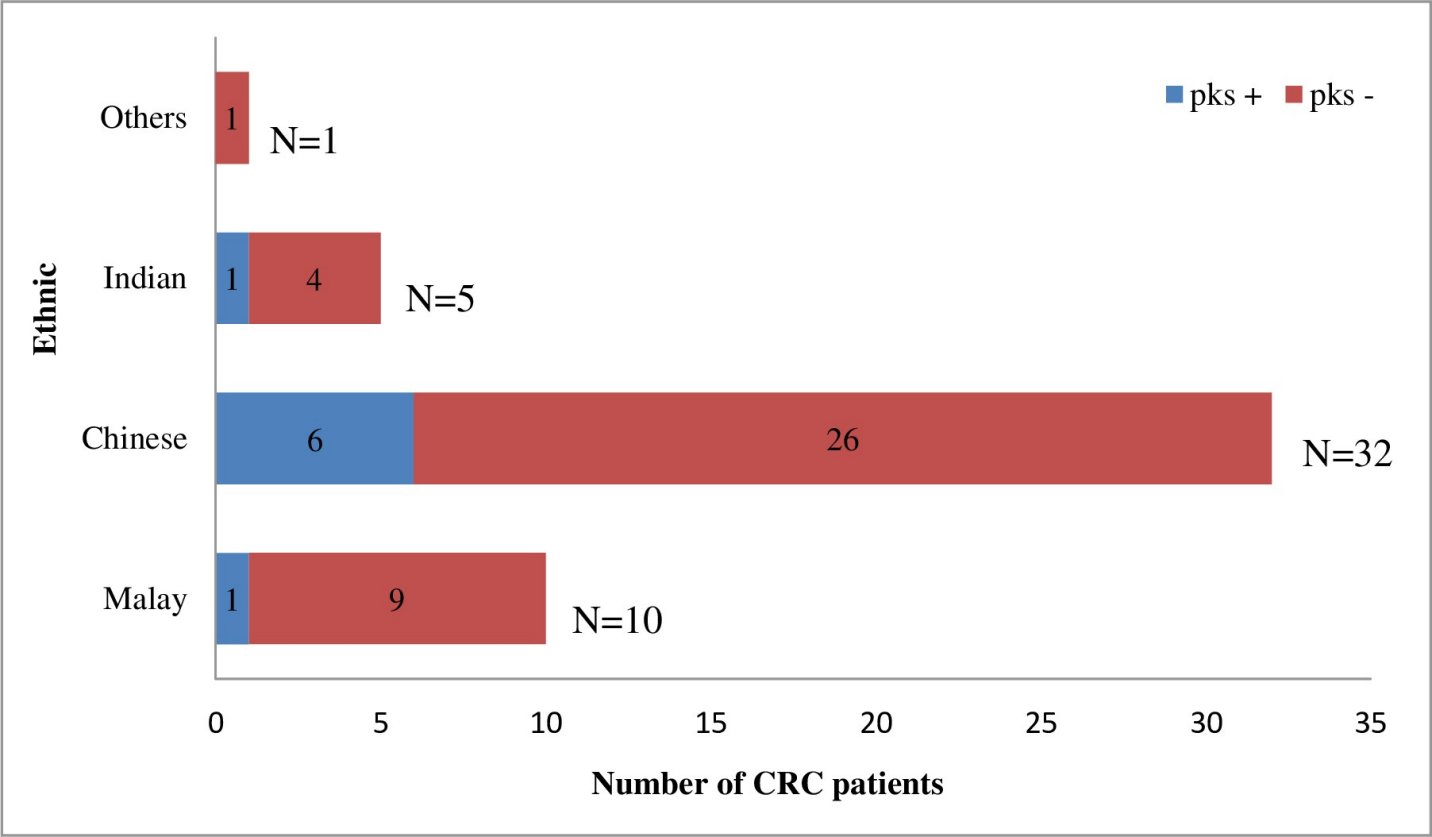
Presence and absence of *pks*^+^ *E*. *coli* within the different ethnic group in colorectal cancer patients.

Approximately five CRC patients with *pks*^+^ *E*. *coli* were diagnosed with an early stage cancer (Stage I/II), while three were diagnosed with the late stage of cancer (Stage III/IV) (Fig 1B). In addition, six out of 32 (18.8%) patients from Chinese ethnicity were *pks*^+^ *E*. *coli* as compared to two (12.5%) from the other two ethnic groups (Fig 3).

### 5.4 Cytopathic effect induced by *pks*^*+*^ *E*. *coli* using primary colon epithelial (PCE) and colorectal carcinoma (HCT116) cell lines

**Fig 4:**
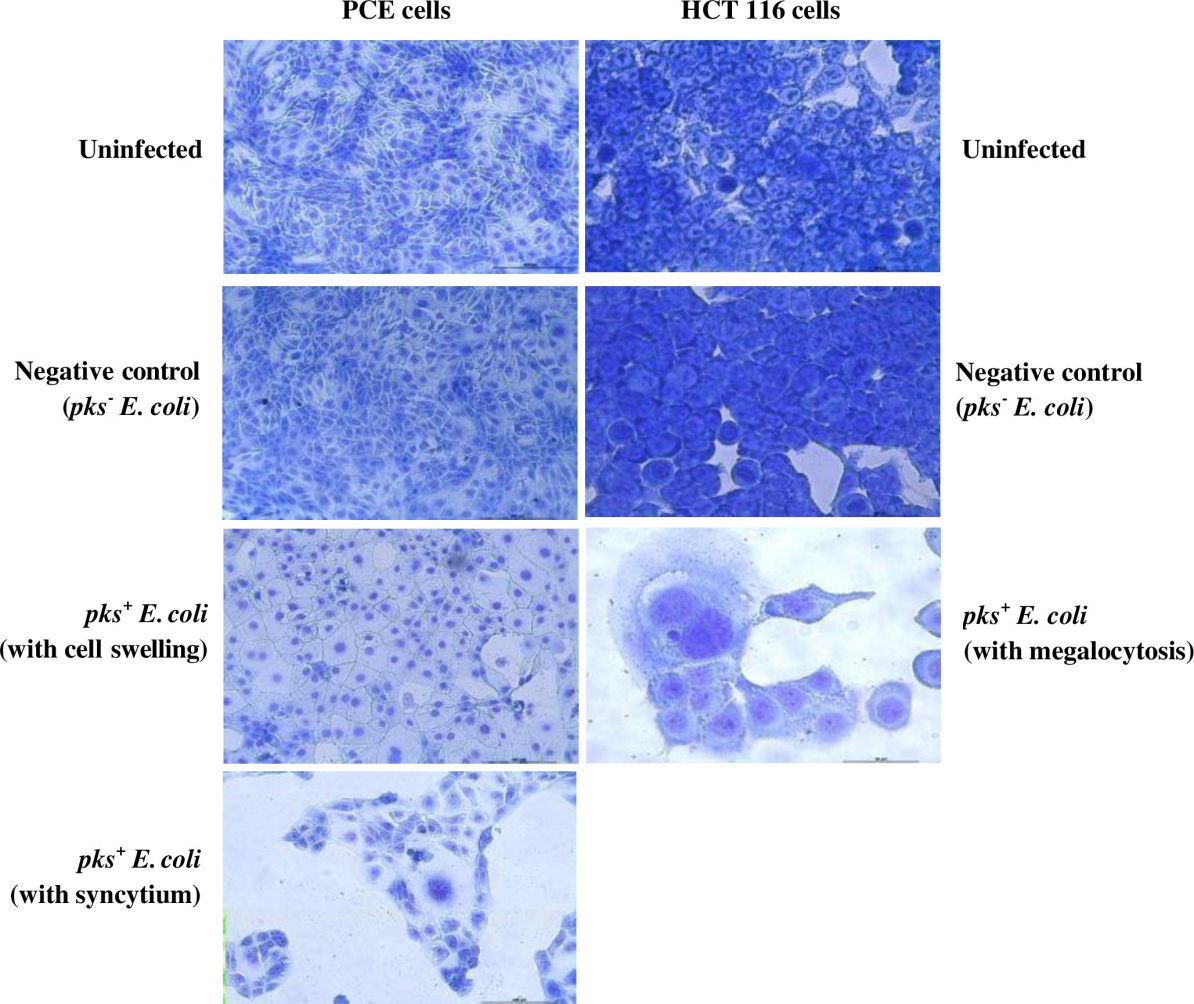
Cytopathic effect induced by *pks*^+^ *E*. *coli* for both PCE and HCT116 cell lines.

The PCE cells infected with *pks*^+^ *E*. *coli* strains isolated from both tumour and their matching non-malignant tissue exhibited cell swelling for three CRC patients while four patients exhibited formation of syncytium post-infection. The *pks*^*+*^ *E*. *coli* strains isolated from either tumor or matching normal colon tissue of only one CRC patient exhibited both syncytium and cell swelling. HCT116 cells infected with *pks*^*+*^ *E*. *coli* isolated from all 8 CRC patients showed megalocytosis with enlarged nuclei and increased cytoplasm being evident (Table 2; Fig 4). *pks*^*-*^ *E*. *coli* however, did not induce CPE in either of the examined cell line.

**Table 2:** Different cytopathic effect of PCE and HCT116 cell lines infected with *pks*^*+*^ *E*. *coli*.

Sample	PCE cells		HCT 116 cells
	Swelling	Syncytium	Megalocytosis
**H2**_**NR**_	-	/	/
**P4**_**N**_	-	/	/
**P4**_**T**_	-	/	/
**P13**_**N**_	/	-	/
**P13**_**T**_	/	-	/
**P17**_**N**_	/	-	/
**P17**_**T**_	/	-	/
**P21**_**N**_	-	/	/
**P21**_**T**_	-	/	/
**P24**_**N**_	/	-	/
**P24**_**T**_	/	-	/
**P27**_**N**_	/	-	/
**P27**_**T**_	-	/	/
**P43**_**N**_	-	/	/
**P43**_**T**_	-	/	/
**P45**_**N**_	-	/	/
**P45**_**T**_	-	/	/

Abbreviation: H, healthy control; NR, proximal normal colon; P, CRC patients; N, non-tumor; T, tumor.

## 6 Discussion

On a global scale, CRC is one of the most common types of cancer and different approaches are being utilized to identify the contributing factors involved in the rise of CRC. The focus of this study was to investigate the possible association of *pks*^*+*^ *E*. *coli* toxin in the pathogenesis of CRC, based on previous reports.

In this study, *pks*^*+*^ *E*. *coli* was found to be more common in CRC patients (14.7%) as compared to normal healthy patients (4.3%). This finding is in agreement with previous studies wherein *pks*^*+*^ *E*. *coli* was over-represented in CRC patients as compared to those without cancer [6], [13], [18], [19]. In addition, we demonstrated that *pks*^*+*^ *E*. *coli* was found in both tumor and matching non-malignant tissue in CRC patients. This statement was supported by other study which stated that within a given patient, the bacterial communities’ adherent to colonic mucosa showed little variation, and the same type of bacteria are found along the length of large intestine [20]. Moreover, it is quite common to have the same clonal *E*. *coli* present in both tumor and tumor-free mucosa of the colon [12].

In this study, six out of eight *pks*^*+*^ *E*. *coli* CRC patients had their tumors on the distal part of the colon. Similar outcome was reported in which *pks*^*+*^ *E*. *coli* was found to more often colonize the distal colon as compared to the proximal colon [13]. This might be due to the qualitative and quantitative difference(s) of the bacterial flora composition along the colon and/or biologic properties of these mucosae that is possibly related to the embryologic origin in which the distal colon develops from the hindgut whereas the proximal colon, from midgut. These differences in origin were reported to lead to different epithelial metabolism, whereby the distal and proximal colon utilizes butyrate and acetate, respectively.

We also observed a slight increase in the frequency of *pks*^*+*^ *E*. *coli* isolation in those with stage II cancer (29.4%) as compared to both stage III and IV CRC combined (27.8%). This is in agreement with another study in which it was reported that there was an increase in the presence of *pks*^*+*^ *E*. *coli* in stage II CRC patients as compared to stage III/IV patients [12]. This difference may be explained by a proposal from Buc et al. that suggests *pks*^*+*^ *E*. *coli* may affect carcinogenesis at different stages of cancer [13]. Although the mechanism is still undefined, in our view, this occurrence may be explained by the involvement of the regional lymph nodes in stages III and IV. At this stage, the affected lymph nodes lose their normal structure and function that can lead to lymphangiogenesis (formation of lymphatic vessels) that may remove unwanted bacteria such as *pks*^*+*^ *E*. *coli* at more advanced CRC stages.

Based on our *in-vitro* infection model, there were obvious changes in the morphology of the primary colon epithelial (PCE) and colorectal carcinoma (HCT116) cell lines upon pre- and post-infection with *pks*^*+*^ *E*. *coli*. The CPE observed in PCE were syncytia (cell fusion) and cell swelling while megalocytosis was observed in HCT116. Each of these CPE are distinct; syncytia are defined as large cytoplasmic masses containing many nuclei that are formed due to the fusion of infected cells, while megalocytosis is the enlargement of both the nucleus and cytoplasm [21], [22]. The link between cytopathic effect (CPE) and carcinogenesis was introduced by one of the initial studies conducted in 1945 that reported the presence of abnormal cells with alterations in the shape as well as the size as compared to normal cells [23]. These changes in the morphology of the cells made the researchers to consider the transformation of the normal cells to become malignant cells due to the presence of certain carcinogen [23]. This process of cell transformation in an *in-vitro* model due to bacteria-host cell interaction represents a crucial advance in the field of cancer research in which the changes may directly represent DNA damage and was designed to identify the abnormal cells formed following exposure to a carcinogenic agent (including bacteria).

For PCE, the changes in the morphology due to infection with *pks*^*+*^ *E*. *coli* might represent the capacity of the specific genotoxin to initiate the process of tumorigenesis. Studies have demonstrated that signs of tumorigenesis such as genomic instability, chromosome aberrations, increased mutation frequency and hallmarks of cellular senescence were observed by the surviving cells upon exposure to *pks*^*+*^ *E*. *coli* [22]. These findings have been identified in an *in-vitro* growth model of tumour cells, perhaps similar to findings observed in CRC patients [22]. Other studies have also showed that the presence of *pks*^*+*^ *E*. *coli* will induce the process of senescence of the normal colonic epithelial cells, and this occurrence may in-turn promote tumor growth through production of growth factors by the senescent cells [24]. Other studies have confirmed the significance of *pks*^*+*^ *E*. *coli* in CRC patients and have concluded that bacteria-host cell contact can lead to gene mutation due to the activation of signaling pathways that are induced by DNA damage [10], [14], [24, 25]. These results may indicate that the presence of *pks*^*+*^ *E*. *coli* may be a risk factor contributing to the formation of tumorigenesis.

Meanwhile, for HCT 116 cell line, the CPE observed may demonstrate that the genotoxin from *pks*^*+*^ *E*. *coli* might also be responsible for the exacerbation of tumorigenesis, rather than just the initiation process. Previous study observed that direct infection of fibroblast cells with *pks*^*+*^ *E*. *coli* promoted cancer cell proliferation and expansion [22]. In order to further confirm the association between *pks*^*+*^ *E*. *coli* and CRC, a study using an *in vivo* model was performed by Arthur et al. whereby they mono-associated mice with *pks*^*+*^ *E*. *coli* with AOM (azoxymethane—a known carcinogen) treatment [25]. Combined AOM treatment and the presence of the bacteria resulted in the presence of invasive carcinoma with high grade dysplasia. Overall, these studies clearly suggest that *pks*^*+*^ *E*. *coli* might be involved in exacerbating tumorigenesis. Although this results does not completely confirm the association of *pks*^*+*^ *E*. *coli* and the initiation and/or exacerbation of tumorigenesis, we could only state that the CPE produced is merely a preliminary result that requires further study and the different type of CPE obtained may be due to activation of different metabolite and/or pathway that ultimately lead to either initiation of tumorigenesis or exacerbation of tumorigenesis.

Although there are many research conducted with regards to *pks*^*+*^ *E*. *coli*, there have been no study thus far that confirmed the presence of specific target site aimed by *pks*^*+*^ *E*. *coli*. However, it has been documented by previous study that the process of pathogenesis due to *pks*^*+*^ *E*. *coli* may occur as follows. Initially, there must be direct contact between the live bacteria with the relevant cells to induce the toxicity of *pks*^*+*^ *E*. *coli* [18]. This *pks* toxin, *pks*^*+*^ *E*. *coli*, has the ability to adhere and invade the colonic epithelial cell through a receptor known as carcinoembryonic antigen-related cell adhesion molecule 6 (CEACAM6) [18]. *pks*^*+*^ *E*. *coli* will later release its toxin to the surrounding environment leading to the changes in the morphology of cells that can be observed as CPE that will cause premature cellular senescence and tumorigenesis [22]. Cells that undergo senescence due to the presence of carcinogen have the ability to overproduce senescence-associated secretory phenotype (SASP) such as growth factors as a by-product upon exposure to *pks*^*+*^ *E*. *coli*. This abnormal overproduction of growth factors will bind to the receptor of the signalling pathways that will subsequently initiate the cascade effect of the pathway and lead to tumorigenesis [24, 26].

As for the presence of more *pks*^*+*^ *E*. *coli* in distal colon as compared to proximal colon, this may be due to the difference in the embryologic origins as distal colon derives from hindgut while proximal colon derives from midgut [27]. This dissimilarity in the origin might influence the characteristics of the epithelial cells or the predisposition towards certain carcinogen. Moreover there were higher presence of allelic losses on chromosome 5, 17 and 18 for distal tumours as compared to proximal tumours. These absences will lead to zero production of certain protein that may play a role in the prevention of carcinogenesis.

In conclusion, we demonstrated that *pks*^*+*^ *E*. *coli* is more commonly isolated from the tumor and normal colon mucosa in Malaysian CRC patients being treated at UMMC as compared to the healthy controls. Additionally, all the *pks*^*+*^ *E*. *coli* isolates from Malaysian CRC patients showed CPE in two distinct cell lines *in-vitro*, suggesting that these bacteria might play a role in the initiation as well as promotion of carcinogenesis. These findings may perhaps represent only one additional factor potentially contributing to colon carcinogenesis in Malaysia. There were limitations in this study such as the insufficient number of samples that led to inadequate comparison between CRC patients and normal healthy patients. Thus, in addition to assessing specific bacteria and the microbiome, future Malaysian studies should focus on the role of dietary intake and biological factors in a more balanced ratio of CRC patients and normal healthy patients together with an *in-vivo* animal model to further assess the factors contributing to CRC and the colon carcinogenicity of Malaysian strains of *pks*^*+*^ *E*. *coli*.

## Supporting information

S1 Appendix Questionnaire; (A) In Bahasa Malaysia (B) In English.
(PDF)

S1 Table Cell confluency for both primary colon epithelial cell line and colorectal carcinoma cell line at 0, 24, 48 and 72 hours. (PDF)

S2 Table Presence of *pks*^*+*^ *E*. *coli* in all the study subjects (healthy controls and colorectal cancer patients). (PDF)

S1 Raw Images PCR amplification. (PDF)

## 7 Abbreviations

clb: colibactin
CPE: cytopathic effect
CRC: colorectal cancer
GI: genomic island
HCT116: colorectal carcinoma cell line
MOI: multiplicity of infection
PCE: primary colon epithelial cell line
pks: polyketide synthetase
