# Supplementary material for: Prevalence and association of pks+ Escherichia coli with colorectal cancer in patients at the University Malaya Medical Centre, Malaysia
Source: PLoS One. 2020 Jan 28;15(1):e0228217. doi: 10.1371/journal.pone.0228217 (PMC6986756; doi:10.1371/journal.pone.0228217)
Supplement: S1 Appendix — Questionnaire; (A) In Bahasa Malaysia (B) In English. (PDF) [file pone.0228217.s001.pdf]

Supplementary Material

S1 Appendix. Questionnaire; (A) In Bahasa Malaysia (B) In English

A)

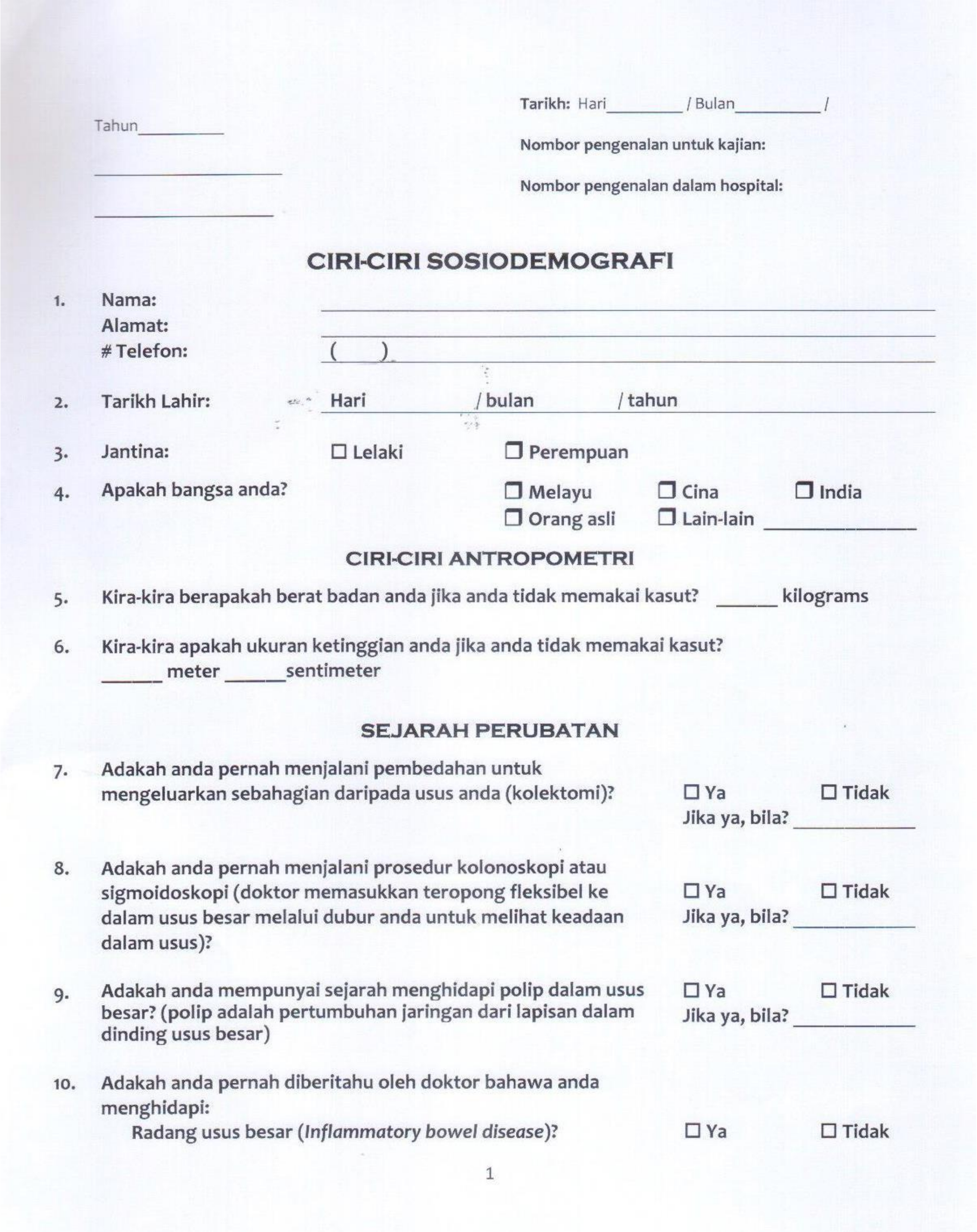

Tarikh: Hari________ / Bulan__________ / Tahun__________

Nombor pengenalan untuk kajian: ____________________

Nombor pengenalan dalam hospital: ____________________

CIRI-CIRI SOSIODEMOGRAFI

1. Nama: ____________________
   Alamat: ____________________
   # Telefon: ( ) ____________________

2. Tarikh Lahir: Hari / bulan / tahun

3. Jantina: ☐ Lelaki ☐ Perempuan

4. Apakah bangsa anda? ☐ Melayu ☐ Cina ☐ India ☐ Orang asli ☐ Lain-lain ______________

CIRI-CIRI ANTROPOMETRI

5. Kira-kira berapakah berat badan anda jika anda tidak memakai kasut? ______ kilograms

6. Kira-kira apakah ukuran ketinggian anda jika anda tidak memakai kasut?
   ______ meter ______sentimeter

SEJARAH PERUBATAN

7. Adakah anda pernah menjalani pembedahan untuk mengeluarkan sebahagian daripada usus anda (kolektomi)? ☐ Ya ☐ Tidak
   Jika ya, bila? ____________

8. Adakah anda pernah menjalani prosedur kolonoskopi atau sigmoidoskopi (doktor memasukkan teropong fleksibel ke dalam usus besar melalui dubur anda untuk melihat keadaan dalam usus)? ☐ Ya ☐ Tidak
   Jika ya, bila? ____________

9. Adakah anda mempunyai sejarah menghidapi polip dalam usus besar? (polip adalah pertumbuhan jaringan dari lapisan dalam dinding usus besar) ☐ Ya ☐ Tidak
   Jika ya, bila? ____________

10. Adakah anda pernah diberitahu oleh doktor bahawa anda menghidapi:
    Radang usus besar (*Inflammatory bowel disease*)? ☐ Ya ☐ Tidak

1

Jika ya, yang mana satu?

Penyakit Crohn's?	☐ Ya	☐ Tidak
Ulcerative Colitis?	☐ Ya	☐ Tidak
Tidak dinyatakan secara khususnya?	☐ Ya	☐ Tidak

11. Adakah anda mengambil ubat untuk merawat penyakit radang usus besar (*Inflammatory bowel disease*)?
☐ Ya ☐ Tidak
Jika ya, apakah ubat-ubat tersebut? ____________

12. Adakah anda pernah diberitahu oleh doktor bahawa anda menghidapi peyakit kanser?
☐ Ya ☐ Tidak
Jika tidak, terus ke soalan nombor 20.

13. Apakah jenis kanser yang anda hidapi dan dimanakah lokasi kanser tersebut?

Jenis kanser	Bulan & tahun diagnosis dibuat	Organ badan yang mempunyai kanser tersebut
____________	____________	____________
____________	____________	____________
____________	____________	____________
____________	____________	____________
____________	____________	____________

14. Adakah anda pernah atau akan menjalani pembedahan sebagai rawatan untuk kanser ini?
☐ Ya ☐ Tidak
Jika ya, bila? ____________
Jika tidak, terus ke soalan nombor 16.

15. Apakah jenis pembedahan tersebut?

____

16. Pernahkah anda menjalani rawatan kemoterapi untuk merawat kanser?
☐ Ya ☐ Tidak
Jika tidak, terus ke soalan nombor 18.

17. Jika anda pernah menjalani kemoterapi,
Bila anda mula menerima rawatan tersebut? ____________
Bila anda habis menerima rawatan tersebut? ____________

18. Pernahkan anda menjalani rawatan radioterapi untuk merawat kanser?
☐ Ya ☐ Tidak
Jika tidak, terus ke soalan nombor 20.

19. Jika anda pernah menjalani radioterapi,
Bila anda mula menerima rawatan tersebut? ______________________
Bila anda habis menerima rawatan tersebut? ______________________

20. Adakah anda sedang menghadapi tanda atau masalah kesihatan seperti cirit birit? ☐ Ya ☐ Tidak

21. Adakah anda sedang /pernah menghadapi tanda atau masalah kesihatan seperti sakit perut? ☐ Ya ☐ Tidak

22. Adakah anda sedang menghadapi tanda atau masalah kesihatan seperti sembelit? ☐ Ya ☐ Tidak

SEJARAH UBAT-UBATAN

23. Sila nyatakan nama ubat serta sukatan atau dos ubat yang anda makan/minum pada setiap hari (atau secara tetap). Contohnya ubat aspirin.

Nama ubat	Sukatan/dos ubat untuk sehari	Tarikh mula ambil ubat tersebut (bulan___/tahun___)

24. Pernahkah anda dirawat dengan ubat antibiotik dalam setahun yang lepas? ☐ Ya ☐ Tidak

Jika ya, bila? ☐ Sekarang ☐ Dalam 3 bulan yang lepas ☐ Dalam 6 bulan yang lepas ☐ Dalam 12 bulan yang lepas
Apakah ubat-ubat tersebut, dan berapakah sukatan dos bagi ubat-ubat tersebut?
____
____

25. Adakah anda mengambil ubat pencair darah dalam satu bulan yang lepas? ☐ Ya ☐ Tidak
Jika tidak, terus ke soalan nombor 27.
Jika ya, sila tanda pada kotak yang sesuai: ☐ Aspirin ☐ Ubat tahan sakit NSAIDs (Ibuprofen, ponstan) ☐ Plavix ☐ Warfarin ☐ Heparin/enoxaparin

26. Jika anda ada mengambil sebarang ubat pencair darah (jawab ya pada soalan 25) bilakah tarikh terakhir anda mengambil ubat tersebut sebelum menjalani sebarang prosedur? ______________________

SEJARAH KELUARGA

Faktor genetik serta ciri-ciri persekitaran adalah factor risiko untuk pertumbuhan kanser. Oleh itu, soalan-soalan berikutnya adalah amat penting untuk memahami hubungan anda dengan ahli keluarga anda.

27. Adakah anda seorang anak angkat? ☐ Ya ☐ Tidak

28. Sila nyatakan berapa orang adik-beradik atau anak yang anda perolehi di ruang yang sesuai dibawah:
Abang/adik lelaki: ______________
Kakak/adik perempuan: __________
Anak lelaki: __________________
Anak perempuan: ______________

SEJARAH KANSER DALAM KELUARGA

29. Sila lengkapkan jadual dibawah bagi setiap orang ahli keluarga (termasuk anak anda) anda yang pernah/sedang menghidapi penyakit kanser. Nyatakan hubungan mereka dengan anda dan tandakan "X" diruang yang sesuai untuk menunjukkan jikalau beliau adalah dari belah ayah atau emak anda. And juga perlu menyatakan tarikh beliau mendapat diagnosis kanser pada kali pertama, dan tarikh meninggal dunia (sekiranya berpatutan).

Hubungan dengan anda	Dari belah ayah atau emak		Tahun lahir	Masih hidup		Tarikh meninggal dunia	Merokok?		Jenis dan lokasi kanser	Umur sewaktu menerima diagnosis
	Ayah	Emak		Ya	Tidak		Ya	Tidak		

4

30. Adakah anda mempunyai ahli keluarga yang pernah menjalani pembedahan untuk mengeluarkan sebahagian dari usus besar mereka? ☐ Ya ☐ Tidak
Jika ya, siapakah beliau? (nyatakan hubungan beliau kepada anda) ______________

PENGGUNAAN TEMBAKAU

31. Adakah anda pernah merokok 100 batang rokok atau lebih sepanjang hidup anda setakat ini? ☐ Ya ☐ Tidak
Jika tidak, terus ke soalan 37

32. Berapakah umur anda sewaktu anda mula merokok secara tetap? ______________

33. Adakah anda masih merokok? ☐ Yes ☐ No

34. Berapa umur anda ketika anda berhenti merokok? ______________

35. Mengambil kira waktu-waktu anda berhenti merokok dan mula merokok semula, berapakah jumlah tahun anda pernah/sedang merokok? ______________

36. Sewaktu anda merokok secara tetap, berapakah jumlah rokok yang anda biasanya hisap dalam sehari? ______________

PENGAMBILAN ALKOHOL/ARAK

37. Adakah anda pernah minum arak, seperti bir, *wine*, *liquor*, etc secara tetap iaitu sekurang-kurangnya sekali dalam satu bulan? ☐ Yes ☐ No
Jika tidak, terus ke soalan 41.

38. Pada umur berapakah anda mula-mula minum arak secara tetap? ______________

39. Sebelum umur 40 tahun, berapa banyak anda minum bir (12 oz.), wine (5oz.), atau liquor (1 oz.) dalam seminggu?
    - nombor bir/wine/liquor seminggu ______________
    - Kurang dari satu dalam satu _ ______________
    - Tidak pernah minum sebelum umur 40 tahun ______________

40. Selepas umur 40 tahun, berapa banyak anda minum bir (12 oz.), wine (5oz.), atau liquor (1 oz.) dalam seminggu? ______________
    - nombor bir/wine/liquor seminggu ______________
    - Kurang dari satu dalam satu ______________
    - Tidak minum selepas umur 40 tahun ______________
    - Masih berumur kurang dari 40 tahun ______________

JENIS PEKERJAAN DAN AKTIVITI FIZIKAL

41. Adakah anda sedang bekerja sekarang?
    ☐ Sedang bekerja ☐ Tidak bekerja ☐ Pesara ☐ Kurang upaya

42. Apakah tahap aktiviti fizikal berkaitan dengan pekerjaan anda pada kebanyakan masa?
    ☐ Activiti ringan (seperti duduk, berdiri, mengangkat objek ringan kurang dari 3kg, dll)
    ☐ Aktiviti sederhana (seperti berjalan atau naik tangga)
    ☐ Aktiviti lasak (seperti bekerja di tapak pembinaan)
    ☐ Tidak bekerja/pesara/kurang upaya

43. Apakah jenis senaman yang anda selalu buat (sekurang-kurangnya 3 kali seminggu)?
    ☐ Kebanyakannya aktiviti sederhana (berjalan pantas, bercucuk tanam, bermain golf, dll)
    ☐ Kebanyakannya aktiviti lasak (berlari, berenang, berbasikal, bermain bolasepak, dll)
    ☐ Tidak bersenam secara tetap

44. Pada umur 20 tahun, apakah jenis senaman yang anda selalunya buat (sekurang-kurangnya 3 kali seminggu)?
    ☐ Kebanyakannya aktiviti sederhana (berjalan pantas, bercucuk tanam, bermain golf, dll)
    ☐ Kebanyakannya aktiviti lasak (berlari, berenang, berbasikal, bermain bolasepak, dll)
    ☐ Tidak bersenam secara tetap

SEKIRANYA AKAN MENJALANI PROSEDUR KOLONOSKOPI ATAU SIGMOIDOSKOPI

45. Prosedur apakah yang anda akan jalani? ☐ sigmoidoskopi ☐ kolonoskopi

46. Mengapakah prosedur tersebut perlu dibuat? ________________________________

47. Apakah jenis persediaan yang anda perlu laksanakan untuk membersihkan usus besar sebelum prosedur ini dijalankan?

B)

Date: Day_____ / Month ______ / Year______
Study ID Number: ____________________
Medical ID Number: ____________________

SOCIODEMOGRAPHIC CHARACTERISTICS QUESTIONNAIRE

1. Name: ____________________
   Address: ____________________
   Telephone #: (   ) ____________________

2. Birthdate: Day / month / year

3. Sex: ☐ Male ☐ Female

4. What is your race or ethnic group? ☐ Malay ☐ Chinese ☐ Indian ☐ Indigenous ☐ Other ____________

ANTHROPOMETRIC CHARACTERISTICS

5. Approximately, how much do you weight, without shoes? ______ kilograms

6. Approximately, what is your height, without shoes? ______ meters ______centimeters

MEDICAL HISTORY

7. Have you had part of your colon extracted (colectomy)? ☐ Yes ☐ No
   If yes, when? ____________

8. Have you ever had a sigmoidoscopy or colonoscopy before? ☐ Yes ☐ No
   If yes, when? ____________

9. Do you have a history of colon polyps? ☐ Yes ☐ No
   If yes, when? ____________

10. Have you ever been told by a doctor that you have
    Inflammatory bowel disease? ☐ Yes ☐ No
    If yes, which one?
    Crohn's disease? ☐ Yes ☐ No
    Ulcerative Colitis? ☐ Yes ☐ No
    Unspecified? ☐ Yes ☐ No

11. Do you take medications to treat inflammatory bowel disease? ☐ Yes ☐ No

1

If yes, what? ____________

12. Have you ever been told by a doctor that you have cancer? ☐ Yes ☐ No
If no, go to question 20

13. What was the name of the cancer(s) or the location in your body?

Cancer	Month & Year Diagnosed	Type of Organ Affected

14. Did you have/ are you scheduled to have surgery for management of this cancer? ☐ Yes ☐ No
If yes, when? __________
If no, go to question 16

15. What kind of surgery did you have or are you scheduled to have?

____

16. Have you received chemotherapy for management of cancer? ☐ Yes ☐ No
If no , go to question 18

17. If you received chemotherapy,
when did you start receiving chemotherapy? ____________
when did you finish with the chemotherapy? ____________

18. Have you received radiotherapy for the management of cancer? ☐ Yes ☐ No
If no, go to question 20

19. If you received radiotherapy,
when did you start receiving radiotherapy? ____________
when did you finish with the radiotherapy? ____________

20. Are you currently having any diarrheal illnesses (not due to bowel prep? ☐ Yes ☐ No

21. Are you currently or have you had abdominal pain? ☐ Yes ☐ No

22. Are you currently having constipation? ☐ Yes ☐ No

2

DRUG HISTORY

23. Please tell us the names and doses of the medications you take on a regular basis, for example aspirin, heart medications, etc.

Name	Dosage per Day	Start Date

24. Have you been treated with antibiotics in the past year? ☐ Yes ☐ No ☐ I don't know

    If yes When? ☐ Currently ☐ Past 3 months ☐ Past 6 months ☐ Past 12months

    What medications & dosage? ____

    ____

25. History of recent (past month) anticoagulant or blood-thinning medications? ☐ Yes ☐ No
    If no, go to question 27
    If yes, please check appropriate box: ☐ Aspirin ☐ NSAIDs (Ibuprofen, Postan) ☐ Plavix
    ☐ Warfarin ☐ Heparin/enoxaparin ☐ Don't know the name

26. If yes question 25, date you stopped taking the medication prior to procedure: ____________________

FAMILY HISTORY

Both genetics and environment could be risk factors for the development of cancer. For this reason, it is important to determine your biological relationship with your family.

27. Are you adopted? ☐ Yes ☐ No

28. How many of each of the following family members do you have?
    Brothers: _________ Sisters: _________ Sons: _________ Daughters: ________

FAMILY CANCER HISTORY

29. Has any of your direct family member had or currently has cancer ☐ Yes ☐ No

If no, go to question 30

If yes , complete the following table for each of your direct family members (including sons and daughters) that have had or currently have cancer. Identify their relationship with you and mark with an "X" if he or she is from the side of your mother or father. You should also specify as accurately as possible age at diagnosis and age of death, if applicable.

Relationship to you	Paternal/ Maternal		Year Born	Still Living		Age at death	Did he/she smoke?		Type or location of cancer	Age at diagnosis
	Father	Mother		Yes	No		Yes	No		

30. **Do you have any family member that has had part of his/her colon extracted (colectomy)?** ☐ **Yes** ☐ **No**

If yes, who? (state his /her relationship with you) ____________

TOBACCO USE

31. **Have you smoked a total of 100 cigarettes or more in your lifetime?** ☐ **Yes** ☐ **No**
If no, go to question 37

32. **How old were you when you first started smoking cigarettes regularly?** ____________

33. **Do you smoke cigarettes now?** ☐ **Yes** ☐ **No**

34. **How old were you when you last quit smoking cigarettes?** ☐ I have never quit

____________ Years old

35. **Considering the times you may have quit smoking and then restarted, how many total years have you or did you actually smoke cigarettes?** ____________________________________

36. During the time you usually smoked regularly, how many cigarettes do or did you usually smoke per day? ____________

ALCOHOL CONSUMPTION

37. Have you ever drunk alcoholic beverages, such as beer, wine, or liquor regularly, that is at least once a month? ☐ Yes ☐ No
If no, go to question 41

38. At what age did you start drinking alcoholic beverages regularly i.e at least once a month? ____________

39. Before the age of 40, how many drinks of beer (12 oz.), wine (5oz.), or liquor (1 oz.) did you usually drink per week?
Number per week ____________
Less than one per ____________
Never drank before age 40 ________

40. After the age of 40, how many drinks of beer (12 oz.), wine (5 oz.), or liquor (1 oz.) did you usually drink per week? ____________
Number per week ____________
Less than one per week ________
Never drank after age 40 ________
Currently aged less than 40 years ________

OCUPATION AND PHYSICAL ACTIVITY

41. What is your current employment status?
☐ Employed/self-employed____☐ Unemployed ☐ Retired ☐ Disabled

42. How would you categorize your weekly physical activity on the job?
☐ Mostly sedentary or light activity (mostly sitting, standing, lifting light objects (less than 3 kilos).
☐ Mostly medium activity (much walking, climbing stairs).
☐ Mostly intense activity (eg. heavy construction work).
☐ Unemployed/retired/disabled

43. What type of exercise do you do regularly (3 times per week)?
☐ Mostly moderate activity (slow walking, gardening, golfing)
☐ Mostly vigorous activity (running, swimming, bicycling, football)
☐ Do not exercise regularly

44. At age 20, what type of exercise did you do regularly (at least 3 times per week)?
- ☐ Mostly moderate activity (slow walking, gardening, golfing etc)
- ☐ Mostly vigorous activity (running, swimming, bicycling, football etc)
- ☐ Did not exercise regularly

IF UNDERGOING COLONOSCOPY OR SIGMOIDOSCOPY

45. What procedure are you undergoing? : ☐ sigmoidoscopy ☐ colonoscopy

46. What are the reasons for the procedure? ____________________

47. What bowel prep did you take? ____________________
