# Supplementary material for: Prevalence and association of pks+ Escherichia coli with colorectal cancer in patients at the University Malaya Medical Centre, Malaysia
Source: PLoS One. 2020 Jan 28;15(1):e0228217. doi: 10.1371/journal.pone.0228217 (PMC6986756; doi:10.1371/journal.pone.0228217)
Supplement: S1 Table — (PDF) [file pone.0228217.s002.pdf]

Supplementary Material

S1 Table. Cell confluency for both primary colon epithelial cell line and colorectal carcinoma cell line at 0, 24, 48 and 72 hours

Infection time (hours)	Type of cell line	
	Primary colon	HCT 116
0	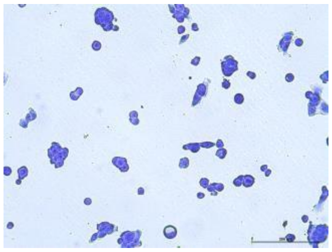	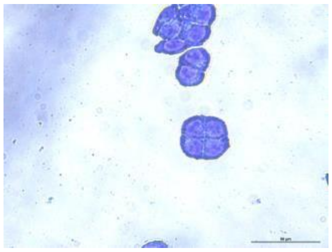
24	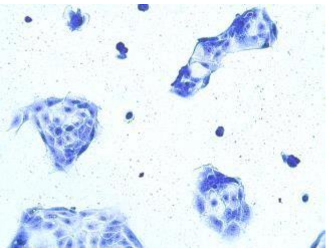	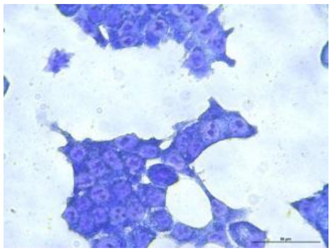
48	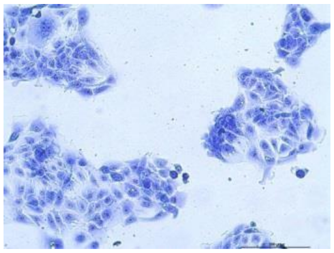	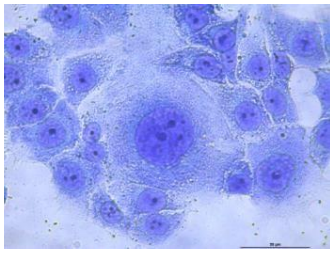
72	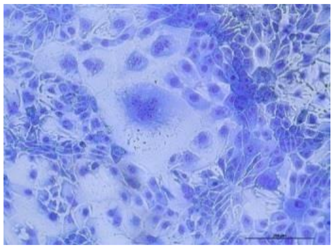	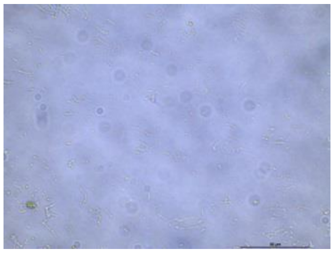
