# Supplementary material for: Prevalence and association of pks+ Escherichia coli with colorectal cancer in patients at the University Malaya Medical Centre, Malaysia
Source: PLoS One. 2020 Jan 28;15(1):e0228217. doi: 10.1371/journal.pone.0228217 (PMC6986756; doi:10.1371/journal.pone.0228217)
Supplement: S2 Table — (PDF) [file pone.0228217.s003.pdf]

Supplementary Material

S2 Table. Presence of *pks*$^{+}$ *E. coli* in all the study subjects (healthy controls and colorectal cancer patients)

Healthy control study ID	Proximal side biopsy	Distal side biopsy	Colorectal cancer study ID	Tumor tissue	Non-tumor tissue
H1	-	-	P1	-	-
H2	√	-	P2	-	-
H3	-	-	P3	-	-
H4	-	-	P4	√	√
H5	-	-	P5	-	-
H6	-	-	P6	-	-
H7	-	-	P7	-	-
H8	-	-	P8	-	-
H9	-	-	P9	-	-
H10	-	-	P10	-	-
H11	-	-	P11	-	-
H12	-	-	P12	-	-
H13	-	-	P13	√	√
H14	-	-	P14	-	-
H15	-	-	P15	-	-
H16	-	-	P16	-	-
H17	-	-	P17	√	√
H18	-	-	P18	-	-
H19	-	-	P19	-	-
H20	-	-	P20	-	-
H21	-	-	P21	√	√
H22	-	-	P22	-	-
H23	-	-	P23	-	-
			P24	√	√
			P25	-	-
			P26	-	-

			**P27**	√	√
			**P28**	**-**	**-**
			**P29**	**-**	**-**
			**P30**	**-**	**-**
			**P31**	**-**	**-**
			**P32**	**-**	**-**
			**P33**	**-**	**-**
			**P34**	**-**	**-**
			**P35**	**-**	**-**
			**P36**	**-**	**-**
			**P37**	**-**	**-**
			**P38**	**-**	**-**
			**P39**	**-**	**-**
			**P40**	**-**	**-**
			**P41**	**-**	**-**
			**P42**	**-**	**-**
			**P43**	√	√
			**P44**	**-**	**-**
			**P45**	√	√
			**P46**	**-**	**-**
			**P47**	**-**	**-**
			**P48**	**-**	**-**
