# Supplementary material for: Prevalence and association of pks+ Escherichia coli with colorectal cancer in patients at the University Malaya Medical Centre, Malaysia
Source: PLoS One. 2020 Jan 28;15(1):e0228217. doi: 10.1371/journal.pone.0228217 (PMC6986756; doi:10.1371/journal.pone.0228217)
Supplement: S1 Raw Images — (PDF) [file pone.0228217.s004.pdf]

A)

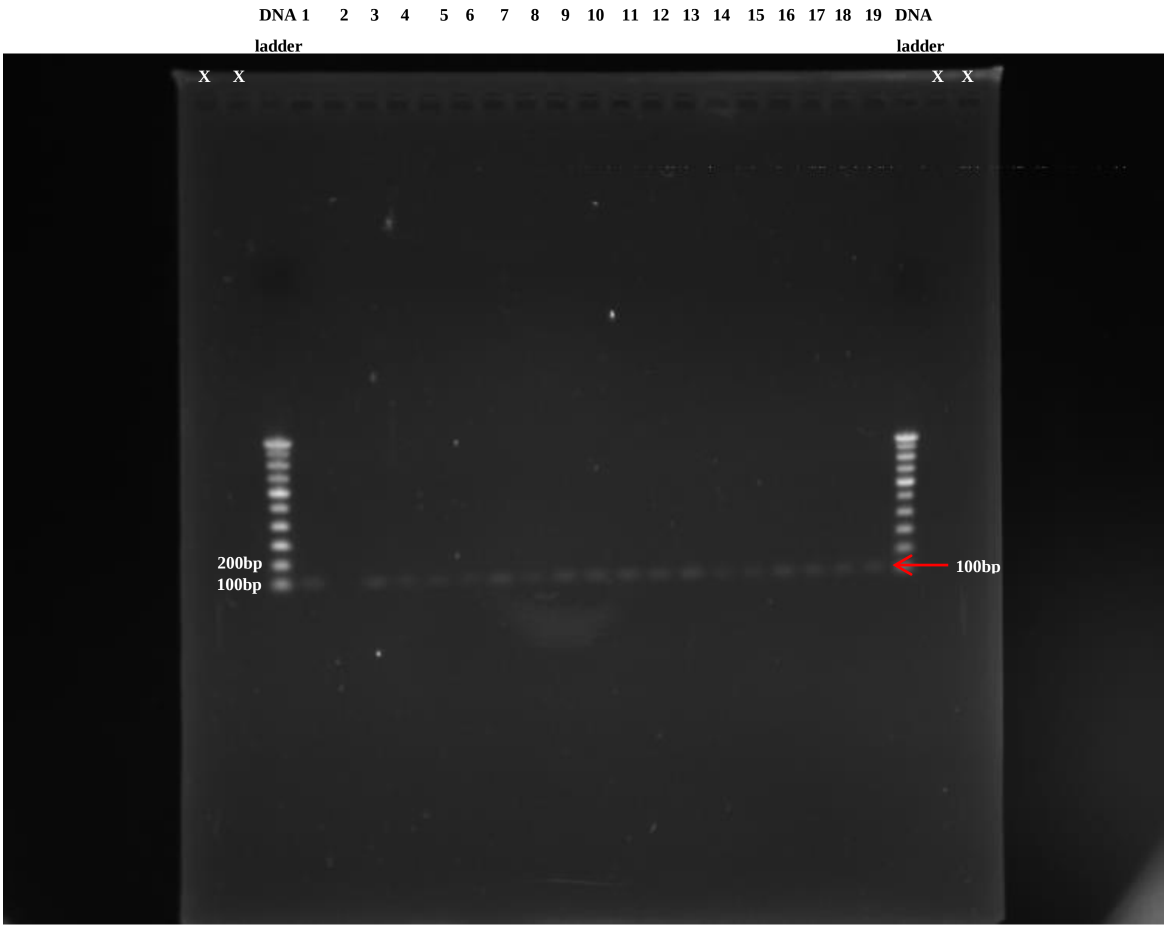

DNA ladder
1 2 3 4 5 6 7 8 9 10 11 12 13 14 15 16 17 18 19
DNA ladder
X X
X X
200bp
100bp
100bp

B)

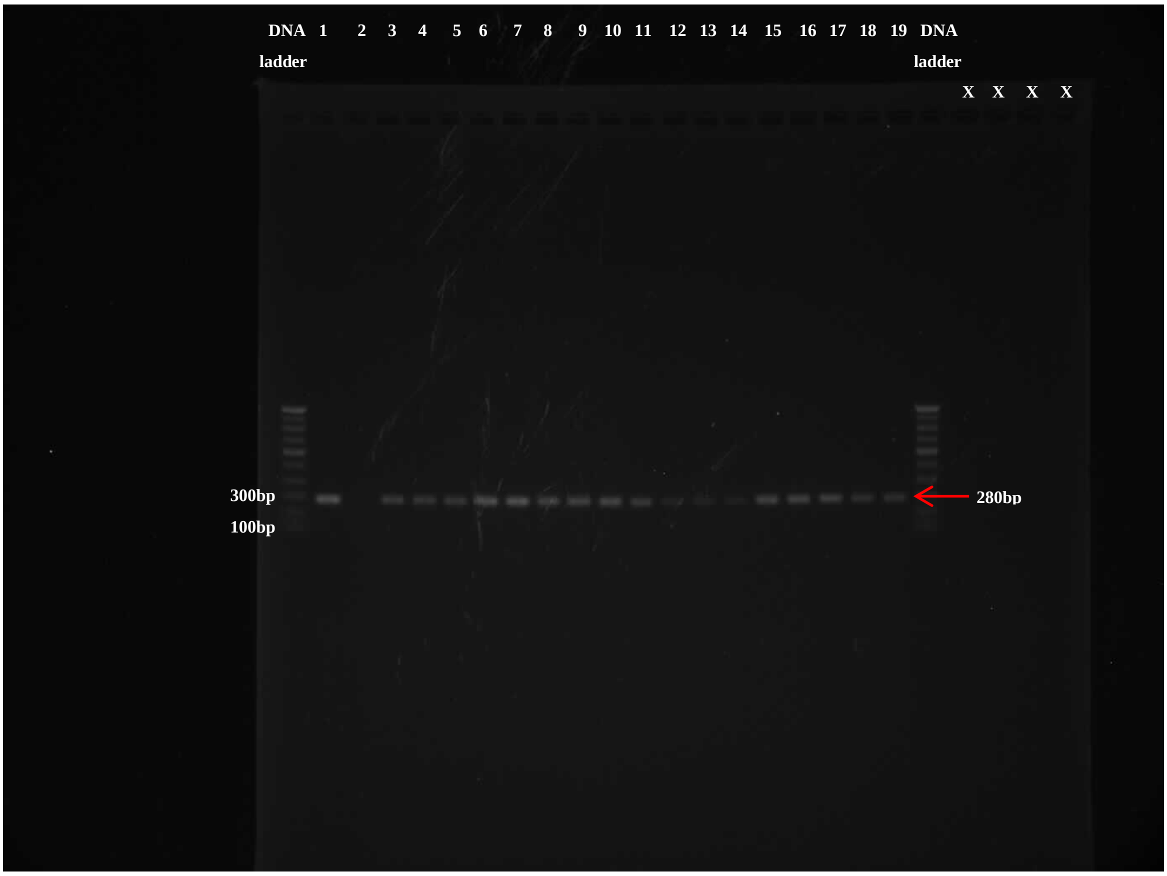

S1_raw_images. PCR amplification; (A) 16*s rRNA* (100 bp) and (B) *colibactin (clbB)* genes (280 bp) of CRC patients and healthy controls was visualized on 1% agarose gel. Lane 1 shows positive control (+) while lane 2 shows negative control (-), followed by lanes 3-18 showing positive bands from CRC patients and lane 19 shows positive band for healthy control.
